# Case report: Incomplete penetrance of autosomal dominant myotonia congenita caused by a rare *CLCN1* variant c.1667T>A (p.I556N) in a Malaysian family

**DOI:** 10.3389/fgene.2022.972007

**Published:** 2023-01-03

**Authors:** Nurul Huda Musa, Karuppiah Thilakavathy, Nur Afiqah Mohamad, Marina L. Kennerson, Liyana Najwa Inche Mat, Wei Chao Loh, Anna Misyail Abdul Rashid, Janudin Baharin, Azliza Ibrahim, Wan Aliaa Wan Sulaiman, Fan Kee Hoo, Hamidon Basri, Abdul Hanif Khan Yusof Khan

**Affiliations:** ^1^ Department of Biomedical Science, Faculty of Medicine and Health Sciences, Universiti Putra Malaysia, Serdang, Malaysia; ^2^ Centre of Foundation Studies, Universiti Teknologi MARA, Cawangan Selangor, Kampus Dengkil, Dengkil, Selangor, Malaysia; ^3^ Genetics and Regenerative Research Group, Faculty of Medicine and Health Sciences, Universiti Putra Malaysia, Serdang, Malaysia; ^4^ Department of Neurology, Faculty of Medicine and Health Sciences, Universiti Putra Malaysia, Serdang, Malaysia; ^5^ Center for Foundation Studies, Foundation in Science, Lincoln University College, Petaling Jaya, Malaysia; ^6^ Northcott Neuroscience Laboratory, ANZAC Research Institute, Sydney Local Health District, Faculty of Medicine and Health, University of Sydney, Sydney, NSW, Australia; ^7^ Molecular Medicine Laboratory, Concord Hospital, Concord, NSW, Australia

**Keywords:** myotonia congenita, *CLCN1*, Thomsen disease, autosomal dominant, chloride channel, case report

## Abstract

Myotonia congenita (MC) is a rare neuromuscular disease caused by mutations within the *CLCN1* gene encoding skeletal muscle chloride channels. MC is characterized by delayed muscle relaxation during contraction, resulting in muscle stiffness. There is a lack of MC case reports and data on the prevalence among Malaysians. We report a clinical case of a 50-year-old woman presents with muscle stiffness and cramp episodes that started in early childhood. She had difficulty initiating muscle movement and presented with transient muscle weakness after rest, which usually improved after repeated contraction (warm-up phenomenon). She was diagnosed with MC after myotonic discharge on electromyography (EMG). Her brother had similar symptoms; however, no additional family members showed MC symptoms. Serum creatine kinase levels were elevated in both the proband and her brother with 447 U/L and 228 U/L recorded, respectively. Genetic analysis by whole-exome sequencing (WES) revealed a previously reported pathogenic *CLCN1* gene variant c.1667T>A (p.I556N). Genetic screening of all family members revealed that the same variant was observed in the children of both the proband and her brother; however, the children did not present with either clinical or electrophysiological MC symptoms. The multiplex ligation-dependent probe amplification (MLPA) analysis conducted identified neither exon deletion nor duplication in *CLCN1*. In conclusion, this report describes the first case of MC in Malaysia in which incomplete penetrance observed in this family is caused by a known pathogenic *CLCN1* variant.

## 1 Introduction

Myotonia congenita (MC) is a chloride channel inherited neuromuscular disorder affecting muscle excitability, resulting in muscle stiffness and varying degrees of muscle weakness (Suetterlin et al., 2014). MC is caused by mutations in the *CLCN1* gene located on chromosome 7q35, which encodes the human skeletal muscle voltage-gated chloride channel (CIC-1) (Pusch, 2002). *CLCN1* mutations cause either autosomal dominant Thomsen disease (OMIM#160800) or autosomal recessive Becker disease (OMIM#255700). These two types of MC can be further distinguished by clinical presentation. Becker disease has more severe symptoms and is frequently associated with transient muscle weakness and muscle hypertrophy in contrast to Thomsen disease, which is less common and usually presented with asymptomatic to moderately severe symptoms (Lossin and George, 2008; Matthews et al., 2010).

To date, more than 318 pathogenic variants (Duno and Vissing, 2021), across the 23 exons or within introns of the *CLCN1* gene have been reported in different populations (Ulzi et al., 2012; Liu et al., 2015; Portaro et al., 2018; Milla et al., 2019). The majority of the mutations are compound heterozygous and associated with Becker disease, with 27 mutations related to Thomsen disease as well as mutations with an undefined inheritance pattern (Morales and Pusch, 2019). *CLCN1* mutations can lead to incomplete or reduced penetrance (Plassart-Schiess et al., 1998). The same *CLCN1* mutation can also be inherited as either a recessive or dominant allele in pedigrees, suggesting that dominance of the allele may contribute to MC’s phenotypic variability (Kubisch et al., 1998; Portaro et al., 2018). To the best of our knowledge, no genetic MC studies have been reported in Malaysia, and the prevalence in this population is still unknown.

The present study was conducted to investigate the clinical and genetic findings of two siblings with MC and their family members. Using the whole-exome sequencing approach, we report a Malaysian patient of Sri Lankan ethnicity presenting with MC with the previously reported p.I556N variant in the *CLCN1* gene classified as likely pathogenic according to ACMG guidelines (Richards et al., 2015).

## 2 Case report

### 2.1 Patient 1

**FIGURE 1:**
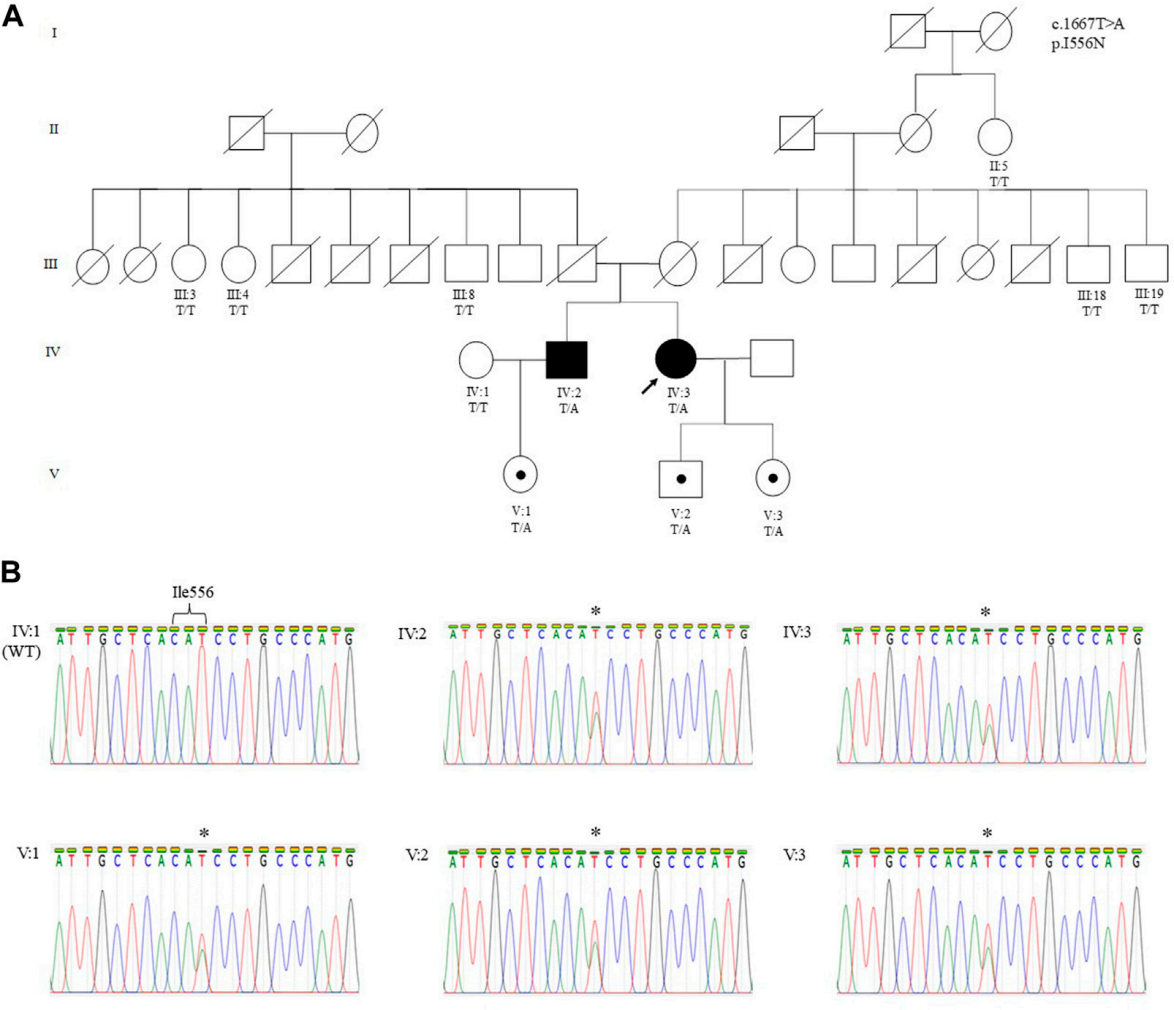
**(A)** Pedigree of a Malaysian family of Sri Lankan Tamil ethnicity with MC showing the segregation of the *CLCN1* c.1667T>A (p.I556N) likely pathogenic variant. The variant is present in Patient 1 (IV:3), Patient 2 (IV:2), and their asymptomatic children. The variant was absent in Patient 2’s wife (IV:1), all paternal (III:3, III:4, and III:8) and maternal (III:18 and III:19) siblings, and maternal great aunt of the proband (II:5). The arrow indicates the proband who underwent WES. Other family members were only genotyped for the specific variant. **(B)** Sanger sequencing electropherograms showing the wild-type (IV:1) and mutant sequence in Patient 1 (IV:3), Patient 2 (IV:2), and their respective children (V:1, V:2, and V:3). The asterisk denotes the heterozygous substitution of T to A. Individuals in each generation of the pedigree are numbered consecutively from left to right. Circles indicate females; squares indicate males. Solid symbols indicate the affected individual with MC, open symbols indicate family members showing a normal phenotype, black dots indicate asymptomatic carriers, and the arrow designates the proband.

The proband (IV:3), a 50-year-old woman, was referred from a primary health clinic for further follow-up of her MC diagnosis. She is of Sri Lankan Tamil ethnicity and is the second born of two siblings from a non-consanguineous marriage (Figure 1). She defaulted clinical follow-up since her early twenties and did not notice any further worsening of her symptoms.

She was clinically diagnosed with MC at the age of 24, after a positive myotonic discharge finding on EMG. The proband did not undergo genetic analysis at the initial diagnosis. Further history disclosed episodes of muscle stiffness (myotonia) and cramps beginning in early childhood at five years old, but these symptoms did not limit her daily activities. She complained of difficulty initiating muscle contraction and mild transient weakness after rest, which improved after exercise (warm-up phenomenon). The myotonia affected all skeletal muscles, including the bulbar muscles, where she also had difficulty swallowing, and myotonia caused cramps and pain in the sternal muscle during sudden sneezing. She had frequent falls when she was younger, especially when she tried to walk after a period of rest. Her pregnancy and history of giving birth (at the age of 34 and 35 years) were unremarkable. She gave birth by Cesarean section *via* epidural anesthesia for both pregnancies and has never been given general anesthesia. There was no extra-muscular manifestation, for e.g., cataracts, endocrine dysfunction, or any previous cardiac problem. The patient was given mexiletine during past intervention but discontinued after three days due to headache. Currently, she is not under any medication except self-care such as practicing yoga.

### 2.2 Physical examination for Patient 1

**FIGURE 2:**
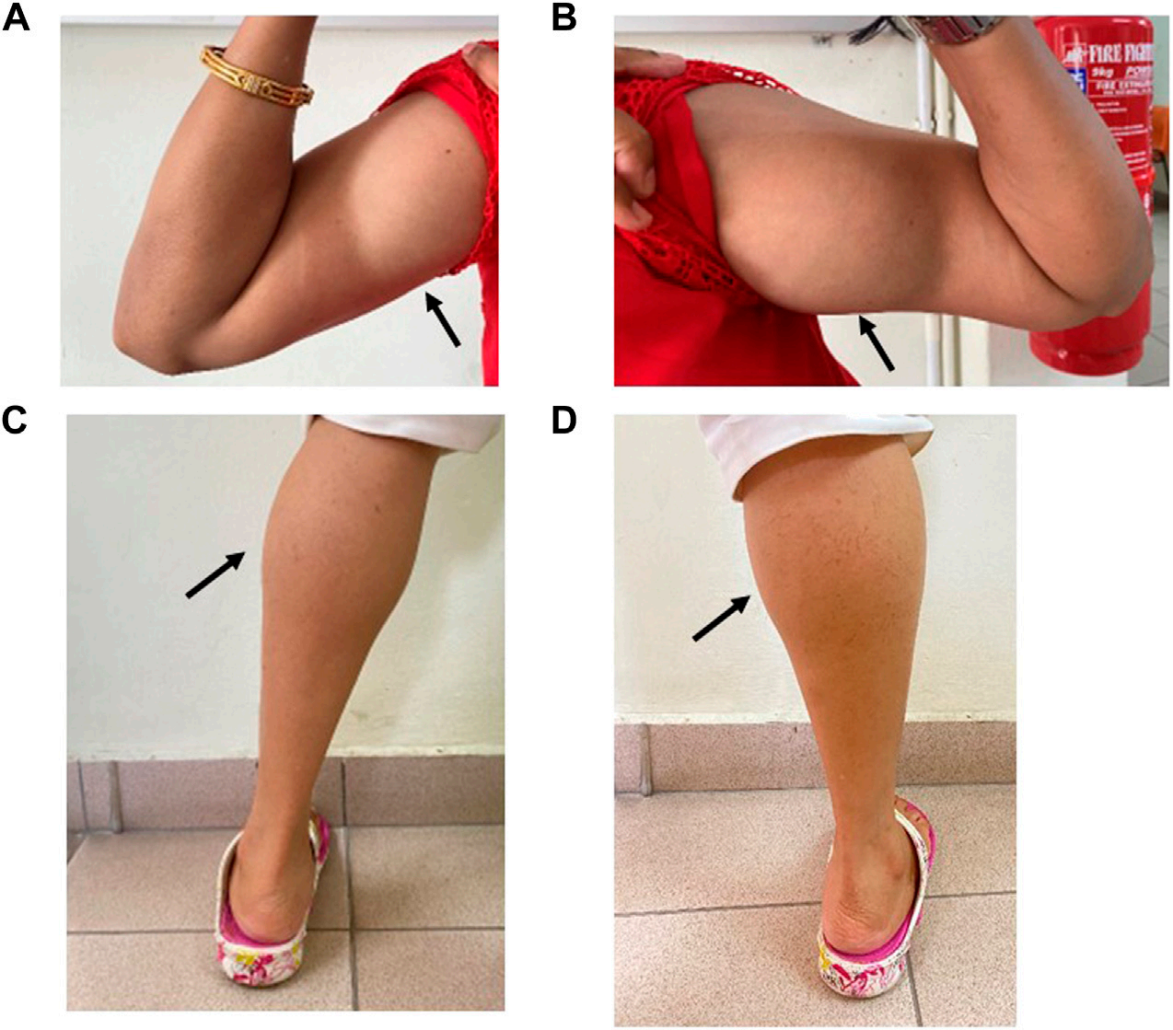
Muscle hypertrophy on the right **(A)** and left **(B)** bilateral biceps and right **(C)** and left calves **(D)** in Patient 1 (IV:3).

Physical examination revealed muscle hypertrophy (athletic appearance) of the bilateral biceps and calves (Figure 2) with a mild myotonic reflex of the hands (percussion and handgrip). The proximal and distal upper and lower limb power was 4 plus out of 5. Gait and sensory examination were normal. Blood investigations were normal apart from the elevated serum creatine kinase level of 447 U/L (normal range < 201 U/L).

**FIGURE 3:**
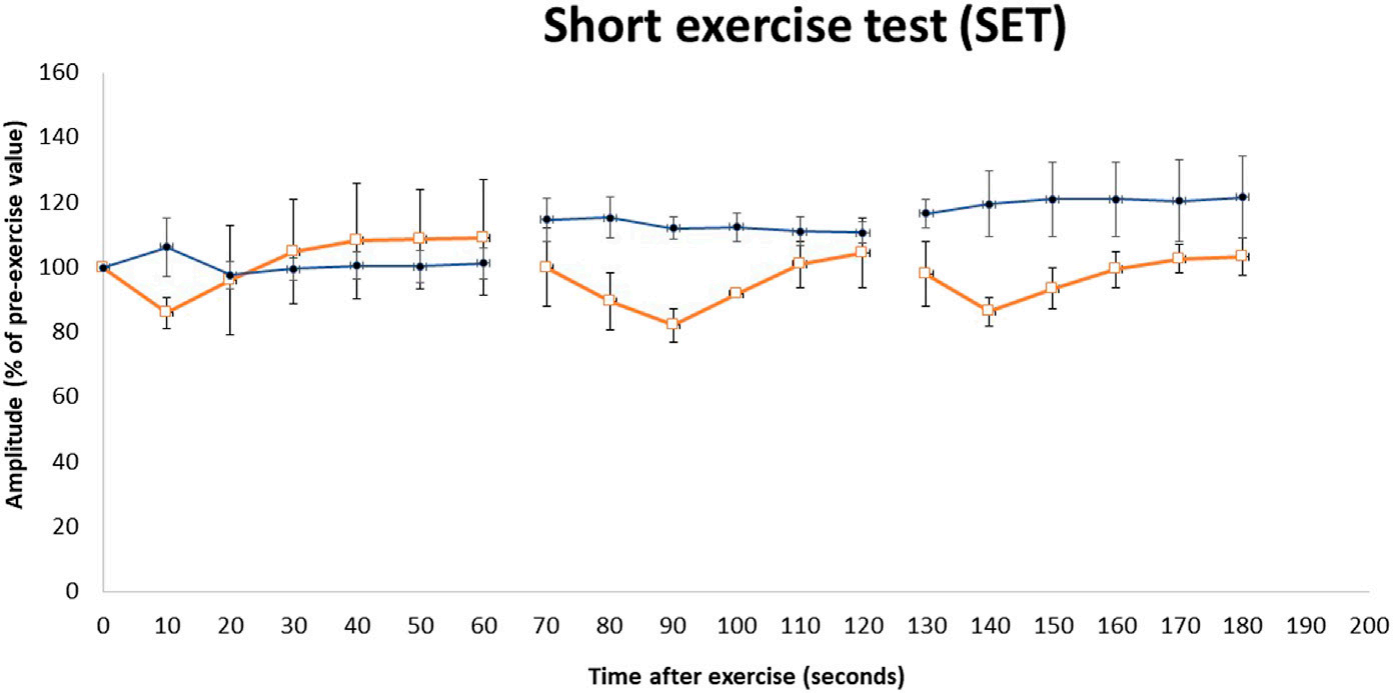
Repeated short exercise test (SET) in MC patients with the c.1667T>A (p.I556N) variant on the *CLCN1* gene. Changes in compound muscle action potential (CMAP) amplitude in the left ulnar nerve following three successive short exercises at room temperature in two heterozygous MC patients (□) and three heterozygous asymptomatic carriers (•). SET shows an initial decrement in CMAP, which subsequently improves (Fournier pattern II) in heterozygous MC patients. The amplitude of CMAP, expressed as a percentage of its value before the trials, is plotted against the time elapsed after the first exercise trials. Symbols and vertical bars represent mean ± SEM.

Nerve conduction studies revealed normal amplitudes, velocity, and latency in the upper and lower limbs. EMG of the first dorsal interosseous, biceps, and tibialis anterior demonstrated an increase in insertional activity with a positive sharp wave and fibrillation coupled with profuse myotonic discharges (dive bomber) with a maximum of 60 Hz. We performed a short exercise test (SET) with serial compound muscle action potential (CMAP), which revealed Fournier pattern II (Figure 3), i.e., initial post-exercise CMAP decrement followed by improvement after 60 s. The SET was also performed after cooling, which showed similar findings at room temperature.

### 2.3 Genomic study

Genomic DNA was isolated from patient’s peripheral blood using QIAamp^®^ DNA Blood Midi Kit (Qiagen, Germany) according to manufacturer’s protocol. Whole-exome sequencing was performed by a commercial company, Macrogen Inc., (Seoul, South Korea). The captured DNA was sequenced as paired-end reads (150 bp) on a NovaSeq 6000 sequencer (Illumina, United States), and exome capture was performed using the SureSelect V5-post kit (Agilent Tech. Ltd., United States). The sequencing reads were aligned to the Human Dec. 2013 (GRCh38/hg38) assembly by using the mapping program BWA. The sequence variant calling and variant filtering were carried out using genome analysis toolkit GATK software, and sequence variants were annotated to dbSNP and SNPs from the 1000 Genome Project using the SnpEff program. The variant calls were obtained from Macrogen in the XLSX file. The Excel file contains thousands of variants including SNVs and indels. Whole-exome sequencing revealed 16 variants in the *CLCN1* gene (2 missense, 3 synonymous, and 11 intronic variants) (Supplementary Table S1). Among these variants, one heterozygous missense variant, p.I556N, was annotated as pathogenic, resulting in the substitution of isoleucine to asparagine at the amino acid 556 position in exon 15. The variant was classified as likely pathogenic according to ACMG guidelines (Richards et al., 2015). The variant was validated using Sanger sequencing (Figure 1) with a pair of primers (I556N-F: 5′-TTA​TTC​CCA​TCC​CAT​CCC​CA-3′; I556N-R: 5′-TAG​AGA​GAG​GGC​TGC​AGG​CT-3′). MLPA analysis was conducted to test for exon deletion and duplication. It was performed using commercially available SALSA MLPA kit P350-C1 *CLCN1*-*KCNJ2* (MRC Holland, Amsterdam, Netherlands), according to manufacturer’s guidelines. The kit contains 24 probes for *CLCN1* (one probe for each exon, two probes for exon 17, two flanking probes, and one upstream and one downstream probe), 4 probes for *KCNJ2*, and 8 reference probes to detect autosomal chromosomal locations. Relative probe signals from each sample were compared to the reference samples (five unrelated individuals without MC) to determine the relative copy number of the target sequences in a sample. Results were analyzed using Coffalyser.Net software (MRC Holland, Amsterdam, Netherlands), and a peak ratio range > 1.25 and < 0.75 was used to determine duplications and deletions, respectively. Results of the MLPA analysis showed that the average probe ratio was within the 0.75–1.25 range demonstrating the absence of exon deletions and duplications of the *CLCN1* gene in the proband, her brother, and all their children (Supplementary Figure S1).

### 2.4 Patient 2

The second patient, a 53-year-old man (IV:2), who is the proband’s older brother, had similar symptoms and the same variant p.I556N. He first noticed muscle stiffness at the age of 13 with hand and leg cramps but remained undiagnosed until the study was conducted. The patient described his symptoms as mild myotonia compared to his sister, had no limitation in daily activities, and could still play sports. He had mild difficulty climbing up the stairs, releasing hand grips on objects, and experiencing eye-closure myotonia.

### 2.5 Physical examination for Patient 2

Physical examination revealed mild muscle hypertrophy of the bilateral biceps and calves. The proximal and distal upper and lower limb power was normal with no myotonic reflex. Gait and sensory examination were normal. Blood investigations were normal apart from the mildly elevated serum creatine kinase level of 228 U/L (normal range < 201 U/L). The nerve conduction study was normal, with EMG showing similar myotonic discharges. SET also showed Fournier pattern II (Figure 3) with no differences noted after cooling.

### 2.6 Other family members

MC symptoms were not reported in the remaining family members of patients 1 and 2, i.e., the deceased parents, the maternal and paternal siblings, and the maternal great aunt (Figure 1). Genetic analysis on available family members showed that the heterozygous p.I556N variant was only present in the children of Patient 1 and Patient 2. EMG was performed on the children, however, the tests did not reveal myotonic discharge (data not shown) in the distal (first dorsal interossei and tibialis anterior) and proximal (biceps brachii and vastus medialis) muscles. Furthermore, the children’s SET results did not show a reduction in CMAP post-exercise (Figure 3), and they all had normal creatine kinase levels (< 201 U/L).

## 3 Discussion

This study reports a proband female patient with a heterozygous p.I556N pathogenic variant in the *CLCN1* gene with mild MC symptoms. The proband’s elder brother presents with similar MC symptoms and carries the heterozygous p.I556N variant. Interestingly, the proband’s deceased parents, maternal and paternal siblings, and children of the proband and her affected brother did not present any MC phenotypic characteristics. However, genetic analysis revealed that all three children from both patients carried the p.I556N variant. In addition, the EMG trials did not reveal any myotonic discharge.

The *CLCN1* gene encodes for the voltage-gated chloride channel in skeletal muscle and plays a vital role in muscle relaxation and action potentials (Pusch, 2002), where overexpression of the gene leads to upregulation of chloride conductance (Lueck et al., 2007). Autosomal dominant Thomsen disease is described by the dominant-negative effect of mutated ClC-1 channel subunits. Dominant variants such as I290M are normally located at one of the monomers residing at the dimer interface or its proximity which cause an effect on the cooperative slow gate. This explains the dominant-negative effect exerted on the unaffected wild-type monomer (Fialho et al., 2007). However, due to the localization, some mutations especially those that localize inside the chloride ion pathway, exhibited no dominant-negative effect, e.g., F484L, which is located in the ion-conducting pore (Skalova et al., 2013; Imbrici et al., 2015). These mutations affect the pore activity directly or induce misfolding of the mutant channel that causes the disease. In contrast, the recessive mutation present in both alleles causes Becker disease affecting the individual fast gate monomer and could also occur due to chloride protein expression in the sarcolemma (Papponen et al., 2008). Moreover, some mutations exhibited both dominant and recessive inheritance patterns which are explained by the defects in both fast and slow gating mechanisms (Weinberger et al., 2012). The *CLCN1* p.I556N missense variant is a T-to-A transition at nucleotide position 1,667 in exon 15 (NM_000083.3) and has been reported to exhibit phenotypic variability (Colding-Jorgensen, 2005). Functional analysis studies of the p.I556N variant showed severe shifts of the voltage dependence; however, co-expression of the mutation with wild-type ClC-1 showed only a slight shift (Kubisch et al., 1998), which explains the milder symptoms in patients with heterozygous variant.

Extensive literature searches for the p.I556N missense variant revealed two other case reports for this variant (Table 1). Both studies reported individuals heterozygous for p.I556N, who typically had mild myotonic symptoms and myotonia discharges (Plassart-Schiess et al., 1998; Arzel-Hezode et al., 2010). Two patients of French descent were identified with the p.I556N variant. One patient present with clinical myotonia showed a decrease in CMAP amplitude after exercise and a consistent short exercise test (SET) results under cold temperature, while the other patient was asymptomatic (Arzel-Hezode et al., 2010). The present study reports similar findings to the heterozygous p.I556N proband that showed mild myotonic symptoms and a decline in CMAP amplitudes after short exercise. The proband’s brother also reported mild symptoms with a later onset at the age of 13.

**TABLE 1:** Previous case studies on myotonia congenita with the *CLCN1* c.1667 T > A (p.I556N) variant.

Author	Type of the study	Subject	Age	Ethnicity	HET/HOM	Sequencing	Clinical evaluation	EMG finding	Additional finding
Plassart-Schiess et al. (1998)	Family case study	Son (proband)	NA	Indian	HOM	Dideoxy-terminator cycle sequencing	Severe myotonic symptoms of early-onset at two years of age with no muscle hypertrophy or muscle weakness	Present with myotonic discharges	First observation of incomplete dominance
		Mother	NA		HET		Mild myotonic symptoms of late-onset at age 20		
		Paternal grandmother	NA		HET		Mild myotonic symptoms of late-onset at age 44	Normal	
		Father	NA		HET		Normal		
Arzel-Hezode et al. (2010)	Family case study	Son (proband)	19	French	HOM	Bidirectional sequencing	Permanent muscle stiffness with very disabling weakness at the initiation of movements after rest	Myotonic discharges in all *CLCN1* I556N carriers	Drastic reduction in CMAP amplitude after a short exercise
		Son (proband)	14		HOM				
		Mother	46		HET		Muscle stiffness with clinical myotonia		Slight decline in CMAP amplitude after a short exercise
		Father	53		HET		Asymptomatic		
Present study	Case report	Proband (female)	50	Sri Lankan	HET	Whole-exome-sequencing	Episodes of myotonia and cramps beginning in early childhood with mild transient weakness after rest	Profuse myotonic discharges	An initial decline in CMAP amplitude after a short exercise with the CK value 447 U/L. Presence of c.352G > T (p.G118W) variant that is classified as benign according to ACMG guidelines
		Brother	53		HET	Sanger sequencing	Muscle stiffness begins at age 13 with hand and leg cramps	Profuse myotonic discharges	An initial decline in CMAP amplitude after a short exercise with the CK value 228 U/L. Presence of c.352G > T (p.G118W) variant that is classified as benign according to ACMG guidelines
		Son	16		HET		Asymptomatic and no myotonic discharge	No myotonic discharges in all *CLCN1* I556N carriers	
		Daughter	15		HET		Asymptomatic and no myotonic discharge	No myotonic discharges in all *CLCN1* I556N carriers	Normal CK value (< 201 U/L). Presence of c.352G > T (p.G118W) variant that is classified as benign according to ACMG guidelines
		Niece	18		HET		Asymptomatic and no myotonic discharge		

CMAP, compound muscle action potential; EMG, electromyography; HET, heterozygotes; HOM, homozygotes; NA, not available.

The p.I556N variant has been reported to show incomplete dominance and reduced penetrance in families (Kubisch et al., 1998; Plassart-Schiess et al., 1998). Plassart-Schiess et al. (1998) initially reported the p.I556N mutation in a family of Indian descent with p.I556N heterozygotes were less affected by MC compared to the homozygote family members. Among three heterozygous individuals in the family, two appeared to have mild myotonia, while another member remained asymptomatic. The phenomenon of reduced dominance in heterozygotes is supported in the present study. Among the five individuals who are heterozygous for the p.I556N variant, two members exhibited mild myotonia, while the other three members are asymptomatic carriers.

Duno et al. (2004) suggested that the influence of differential allelic expression on disease progression may explain the symptomatic variability within dominant myotonic family members. For instance, their study demonstrated that the p.R894X variant exhibits both dominant and recessive inheritance, where the severely affected dominant patient expressed twice the amount of R894X mRNA allele relative to the wild-type allele. The p.F307S mutation found in a family with dominant inheritance supported with electrophysiological characterization (Kubisch et al., 1998) has been subsequently found in the homozygous state in families with recessive inheritance (Pusch, 2002).

From the two previous reports and the present study, the p.I556N variant in the heterozygous individuals showed diverse phenotypic variability ranging from mild myotonia to asymptomatic with myotonic discharge revealed only by EMG or asymptomatic with no clinical symptoms. Several other hypotheses have been proposed to explain the clinical variability, including trans-acting genetic modifiers; epigenetic, hormonal, or environmental factors; and dominance with low penetrance (Suetterlin et al., 2014; Cannon, 2015). Duno et al. (2004) showed that allelic variation may likely contribute to phenotypic diversity; however, the specific mechanism is still unknown and could be based on polymorphisms at the promoter or enhancer regions. Hormonal influence is another factor that had shown to aggravate clinical myotonia, where hypothyroidism unmasked the clinically asymptomatic individual. The myotonic phenotype associated with thyroid disease improved with L-thyroxine replacement rather than treatment with antimyotonic medication (Passeri et al., 2014). Although thyroid hormone-responsive elements are not being found in the *CLCN1* upstream region (Lorenz et al., 1994), it is suggested that the thyroid might have an indirect effect such as changing the chloride internal concentrations. Epigenetics is another factor that could affect the phenotypic variability. DNA methylation as the first layer of a heritable epigenetic mark seems to play a role in preventing gene reactivation together with histone modifications (Feng et al., 2007). It usually occurs at CpG sites (sites where cytosine is followed by guanine) and regions containing high levels of CpG dinucleotides (called CpG Island) localized at or near gene promoter (Illingworth et al., 2010) that can inhibit gene expression (Deaton and Bird, 2011). Nevertheless, there is increasing evidence that revealed an epigenetic modification in neuromuscular disorders playing a role in the pathogenesis of the disease (Buckley et al., 2016; Bennett et al., 2019; Cerrato et al., 2020). Furthermore, pregnancy, temperature changes, fatigue, hunger, emotional stress, and menstruation are among the environmental factors that might increase myotonia (Colding-Jorgensen, 2005). These possible mechanisms, whether acting alone or in combination, may explain why the same mutation causes different degrees of channel dysfunction in different patients (Kubisch et al., 1998), thereby highlighting the variability of the MC phenotype with p.I556N.

The MLPA analysis is a commonly used method to identify exonic deletion and duplication. The severity of the proband’s phenotype resembles a recessive clinical form; therefore, the MLPA analysis was conducted to exclude the coexistence of a rearrangement associated with the p.1556N variant. The analysis revealed no deletion or duplication in the exonic regions of *CLCN1* in the proband. This is similar to the study by Lehmann-Horn et al. (2011), which showed that no *CLCN1* alteration was identified in the coding sequence and the exon–intron boundaries in a Turkish family. In contrast, Rayan et al. (2012) identified exon deletions or duplications in *CLCN1* in 6% of patients with MC.

Next-generation sequencing provides an effective and rapid strategy for detecting genetic variants in humans by enabling all coding exons of the genome to be sequenced in a single test. The present study used whole-exome sequencing approach, which was cost-effective, and allowed detailed analyses of the protein-coding exome to detect common and rare genetic variants. Next-generation sequencing is now routinely used in a diagnostic setting as testing can be designed to perform either targeted or genome-wide capture of human genes to rapidly diagnose a disease (Rabbani et al., 2014). Being currently in its infancy, this technology is rapidly developing and growing as the choice of molecular testing in the Malaysia’s healthcare setting.

## 4 Conclusion

In conclusion, we have identified a rare heterozygous c.1667T>A (p.I556N) pathogenic variant in the *CLCN1* gene as the first reported MC study in Malaysia. The variant showed autosomal dominant inheritance with incomplete penetrance of MC. Understanding the genotype–phenotype correlation and modes of transmission for this variant will shed light on the affected family to predict the risk of the disease transmission and the probability of developing the disease at the age of over 20 years as found by Plassart-Schiess et al. (1998). Knowledge of the pathomechanisms is important for appropriate diagnostic testing, genetic counseling for MC patients, and better treatment, ultimately leading to precision and personalized medicine.

## Data Availability

The datasets for this article are not publicly available due to concerns regarding participant/patient anonymity. Requests to access the datasets should be directed to the corresponding authors.

## Abbreviations

ACMG, American College of Medical Genetics and Genomics; BWA, Burrows–Wheeler Aligner; CMAP, compound muscle action potential; ClC-1, chloride channel; *CLCN1*, chloride voltage-gated channel 1; dbSNP, Single Nucleotide Polymorphism Database; EMG, electromyography; GATK, Genome Analysis Toolkit; Indels, insertion–deletion mutations; MC, myotonia congenita; MLPA, multiplex ligation-dependent probe amplification; SET, short exercise test; SNPs, single-nucleotide polymorphisms; SNVs, single-nucleotide variants; TRE, thyroid hormone-responsive elements; WES, whole-exome sequencing; XLSX, Excel Microsoft Office Open XML format spreadsheet file.
