# Supplementary material for: Case report: Incomplete penetrance of autosomal dominant myotonia congenita caused by a rare CLCN1 variant c.1667T>A (p.I556N) in a Malaysian family
Source: Front Genet. 2023 Jan 3;13:972007. doi: 10.3389/fgene.2022.972007 (PMC9842662; doi:10.3389/fgene.2022.972007)
Supplement: Supplementary file 1 [file Table2.DOCX]

The variant has been uploaded to LOVD (Leiden Open Variation Database) website.

LINK:

<https://databases.lovd.nl/shared/variants/0000812999#00005230>
