# Supplementary material for: Case report: Incomplete penetrance of autosomal dominant myotonia congenita caused by a rare CLCN1 variant c.1667T>A (p.I556N) in a Malaysian family
Source: Front Genet. 2023 Jan 3;13:972007. doi: 10.3389/fgene.2022.972007 (PMC9842662; doi:10.3389/fgene.2022.972007)
Supplement: Supplementary file 2 [file Table3.DOCX]

**Supplementary Figure 1 – MLPA Analysis**

(A)


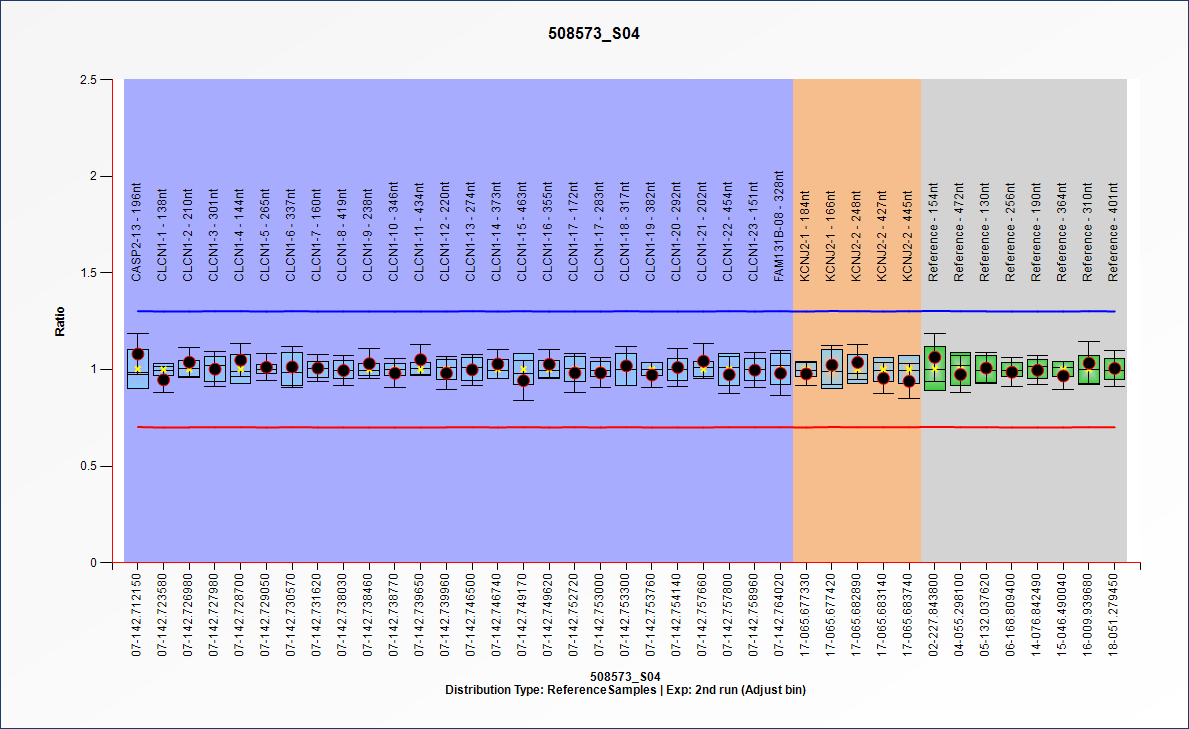


(B)


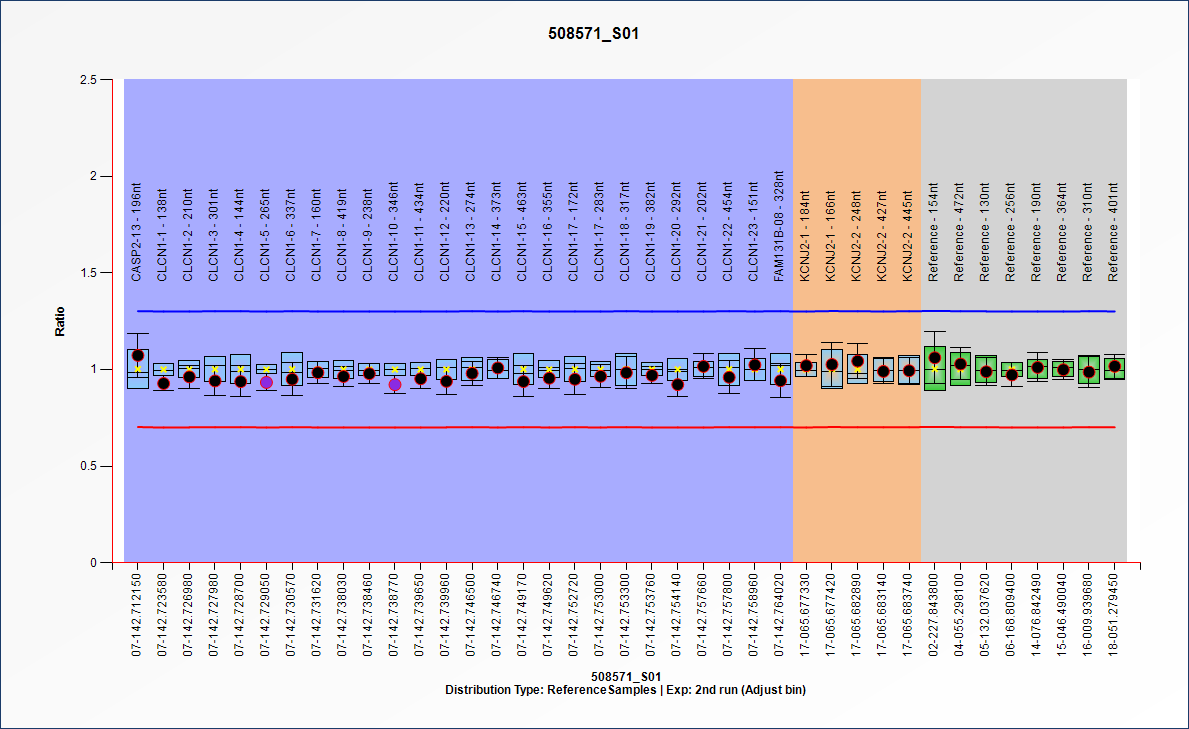


(C)


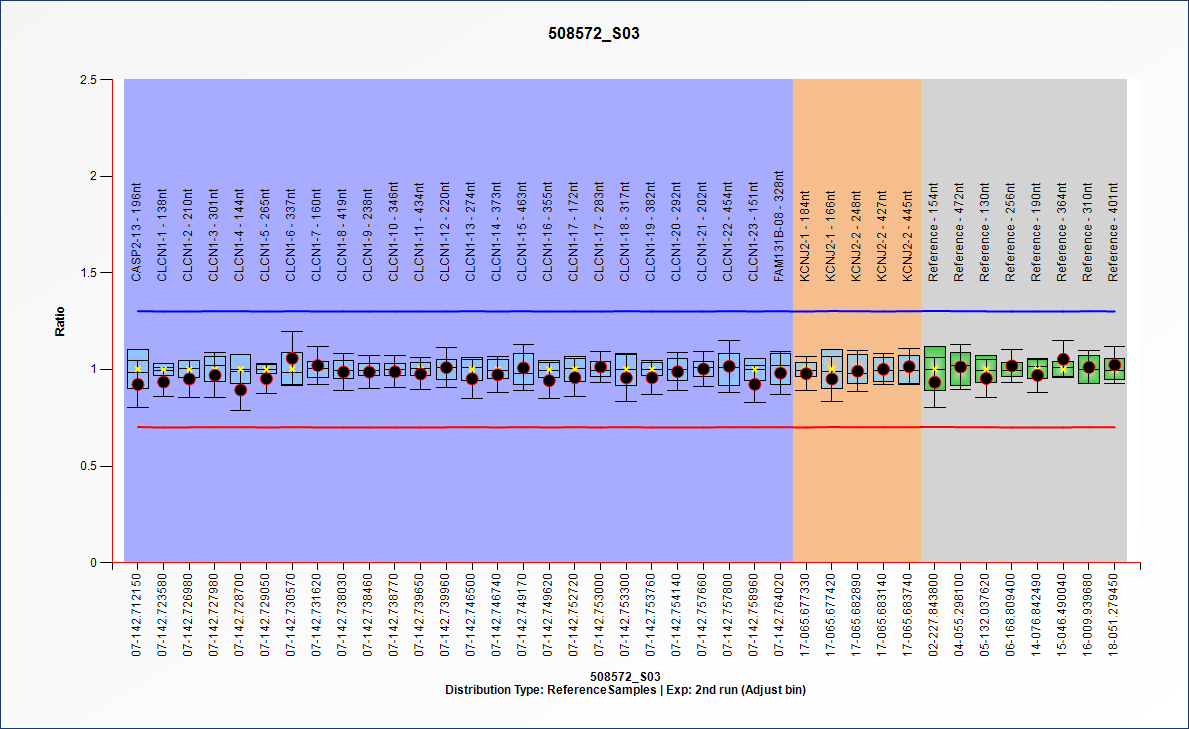


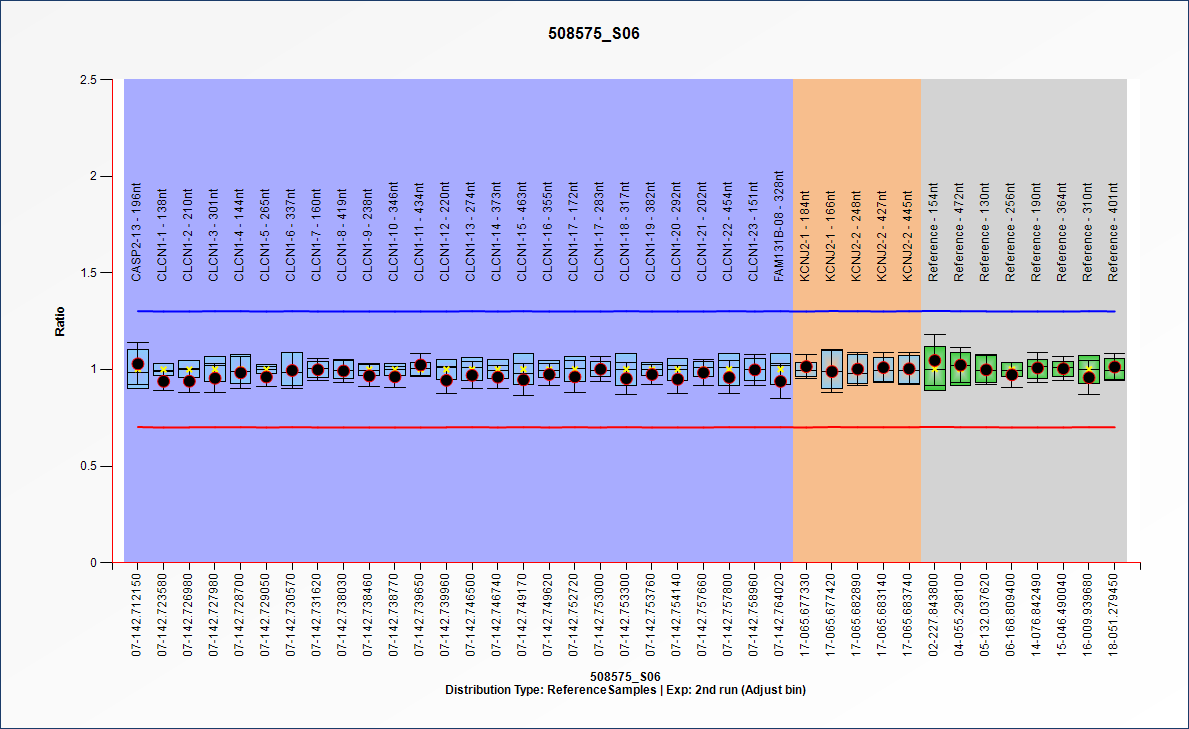


(D)

(E)


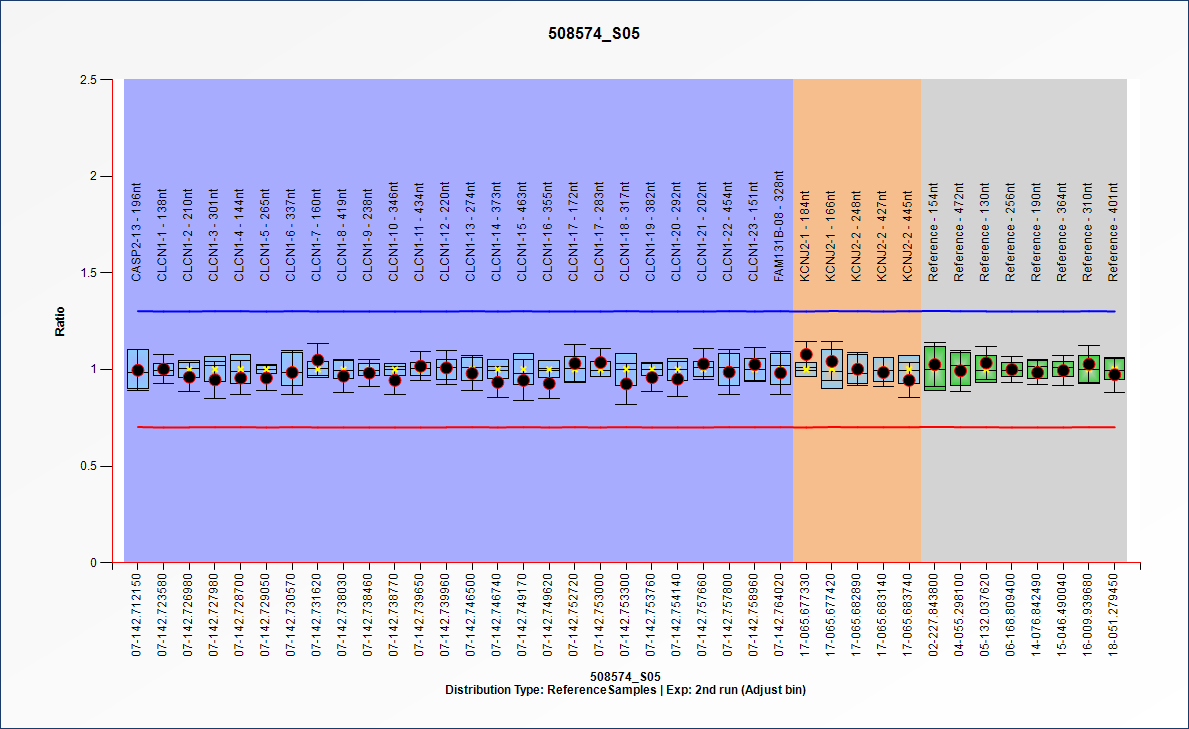


A ratio chart of MLPA result of the patients using SALSA MLPA kit P350-A1 *CLCN1*-*KCNJ2* region probe mix. (A) Proband, IV:3 (B) IV:2 (C) V:1 (D) V:2 and (E) V:3. The black spots represent MLPA probes, the upper blue line indicates a peak ratio of 1.3 and any probes above this line represent a duplication, the lower red line indicates a peak ratio of 0.75 and any probes below this line represents a deletion, and the probes between the two lines are considered as normal two copies. The chart is showing no deletion or duplication as all the spots are between both cut-off lines in the ratio chart. The analysis also contains a *CASP2-13* upstream probe and three exon probes for *KCNJ2*, which are other genes analysed by this kit.

Figure shows the probe ratio of the proband (orange bars) vs reference samples (blue bars) for the 23 exons.
